# Maintenance of Long-Range DNA Interactions after Inhibition of Ongoing RNA Polymerase II Transcription

**DOI:** 10.1371/journal.pone.0001661

**Published:** 2008-02-20

**Authors:** Robert-Jan Palstra, Marieke Simonis, Petra Klous, Emilie Brasset, Bart Eijkelkamp, Wouter de Laat

**Affiliations:** Department of Cell Biology and Genetics, Erasmus MC, Rotterdam, The Netherlands; Institute of Genetics and Molecular and Cellular Biology, France

## Abstract

A relationship exists between nuclear architecture and gene activity and it has been proposed that the activity of ongoing RNA polymerase II transcription determines genome organization in the mammalian cell nucleus. Recently developed 3C and 4C technology allowed us to test the importance of transcription for nuclear architecture. We demonstrate that upon transcription inhibition binding of RNA polymerase II to gene regulatory elements is severely reduced. However, contacts between regulatory DNA elements and genes in the β-globin locus are unaffected and the locus still interacts with the same genomic regions elsewhere on the chromosome. This is a general phenomenon since the great majority of intra- and interchromosomal interactions with the ubiquitously expressed *Rad23a* gene are also not affected. Our data demonstrate that without transcription the organization and modification of nucleosomes at active loci and the local binding of specific trans-acting factors is unaltered. We propose that these parameters, more than transcription or RNA polymerase II binding, determine the maintenance of long-range DNA interactions.

## Introduction

**Figure 1:**
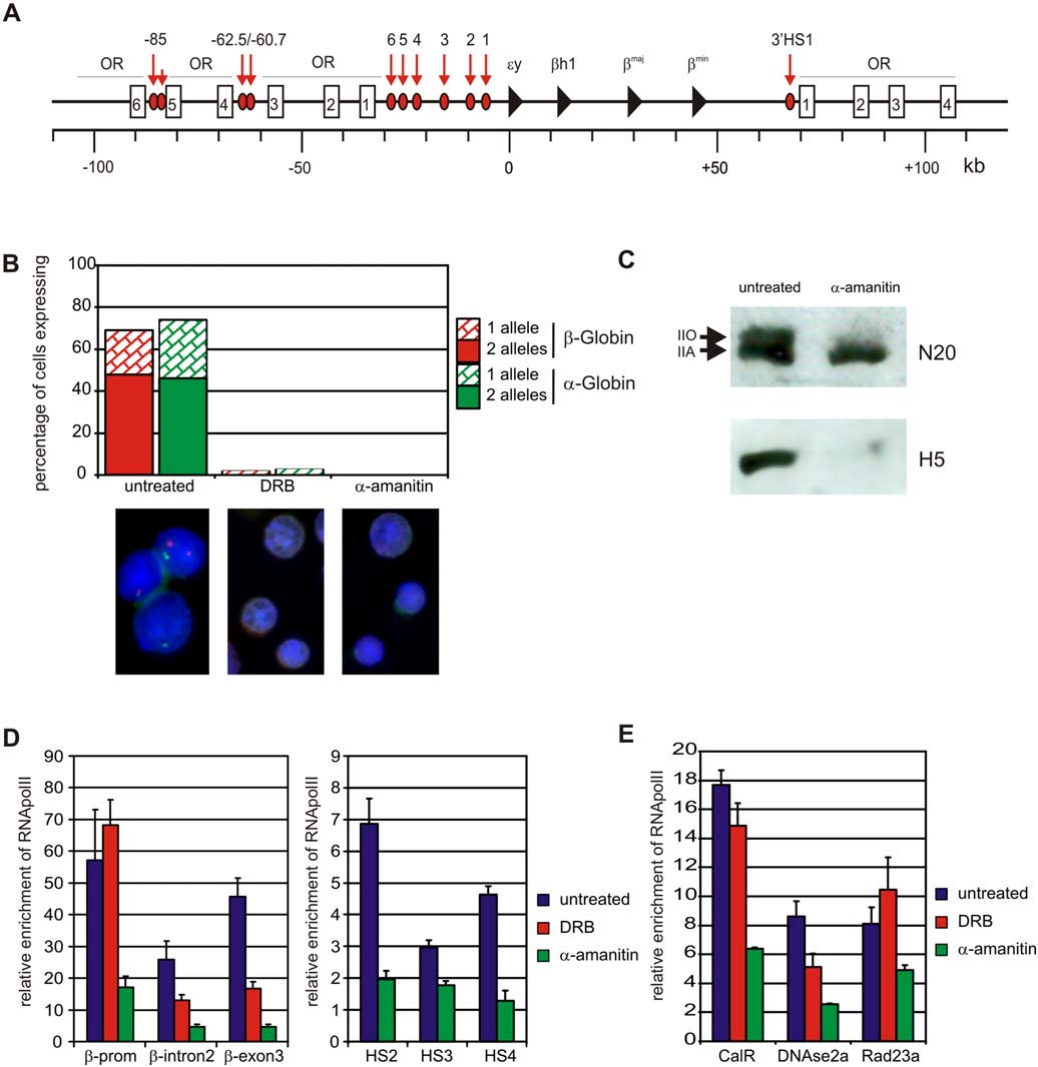
Transcription is efficiently inhibited by DRB and α-amanitin. (A) Schematic presentation of the murine β-globin locus. Red arrows and ellipses depict the individual HSs. The globin genes are indicated by black triangles. The white boxes indicate the olfactory receptor (OR) genes (5′OR1-6 and 3′OR1-4). Distances (roman numerals) are in kb counting from the site of initiation of the εy gene. (B) Primary RNA-FISH of DRB and α-amanitin treated cells. Red bars indicate percentage of cells expressing β-globin and green bars indicate percentage of cells expressing α-globin. Representative examples of images are shown. (C) Reduction of the active elongating form of RNAPII as detected by western blot. Top panel using an antibody against the RPB1 subunit of RNAPII (N20). IIO represent the phosphorylated form of RNAPII, IIA the unphosporylated form. Bottom panel using an antibody against the Ser2 phosphorylated CTD of RNAPII (H5). (D) RNAPII binding at the β-major gene and hypersensitive sites of the LCR. Enrichment is relative to amylase. Blue bars depict untreated samples, red bars DRB treated samples, green bars α-amanitin treated samples. (E) RNAPII binding at regulatory elements within the Rad23a locus upon DRB and α-amanitin treatment. Enrichment is relative to amylase. Blue bars depict untreated samples, red bars DRB treated samples and green bars α-amanitin treated samples. Error bars indicate standard error of mean.

An intricate relationship appears to exist between chromosome folding and gene expression in the mammalian cell nucleus [1], [2]. At the level of gene loci, regulatory DNA elements communicate with target genes located sometimes tens or even hundreds of kilobases away by contacting them, thereby looping out the intervening chromatin fiber. This was shown originally for the mouse β-globin locus, which extends over 180 kb and contains several c*is-*regulatory elements dispersed throughout the locus (Figure 1A). In expressing cells, these regulatory elements cluster with the active genes to form a so-called Active Chromatin Hub (ACH) [3], [4]. This spatial conformation is erythroid-specific and developmentally regulated [5] and depends on several (tissue-specific) transcription factors [6]–[8]. Comparable interactions between genes and *cis*-regulatory elements have been demonstrated for several other gene loci (e.g.[9], [10]).

Large-scale nuclear architecture is also correlated with RNA polymerase II (RNAPII) transcription. For example, clusters of active genes on chromosomes preferentially locate at the edge or outside of their chromosome territory [11], [12]. Moreover, actively transcribed genes tens of mega-bases apart on the chromosome or even on other chromosomes can come together in the nucleus [13], [14]. Recently we have used novel 4C technology to analyze the genomic environments of several gene loci and found that active and inactive loci tend to separate in the nuclear space. The β-globin locus was found to switch its nuclear environment in relation to its expression status, with the active β-globin locus contacting active loci and the inactive β-globin locus contacting inactive loci elsewhere on the chromosome [15]. It is unclear how local interactions between regulatory DNA elements and long-range contacts between active genes are established. It has however been suggested that the process of RNAPII transcription itself plays an important role in shaping the genome [13], [14], [16]–[19].

In this study we tested the prediction made by these models that inhibition of transcription changes DNA folding and eliminates looping [18], [20]. We demonstrate that ongoing RNAPII transcription or the presence of RNAPII at regulatory sites is not required to maintain the shape of the genome in the mammalian cell nucleus. In absence of transcription the overall chromatin state remains unaltered. This observation supports the notion that the organization and modification of nucleosomes along the DNA fiber and the binding of specific *trans*-acting factors, more than transcription or RNAPII-binding, determines the interaction between DNA loci and the formation of chromatin loops.

## Results

### Inhibition of transcription in the β-globin locus

To study the role of RNAPII transcription in nuclear architecture, we investigated the intricate folding of the β-globin locus and its long-range contacts with other genes in primary erythroid cells treated with drugs that inhibit transcription. First, we extensively tested how drug treatment affected transcription at the β-globin locus. Single cell suspensions made from freshly dissected E14.5 fetal livers were cultured for five hours in the presence of very stringent concentrations of α-amanitin (100 µg/ml), a drug that inhibits RNAPII transcription initiation and elongation. Previous studies have demonstrated that under similar conditions RNAPII transcription is inhibited within one hour of treatment while the level of serine2-phosphorylated RNAPII is strongly reduced [21], [22]. In order to distinguish between effects of transcription initiation versus elongation on genome organization we also used 5,6-dichloro-1-β-D-ribofuranosylbenzamidazole (DRB) (3 hours, 100 µM) in some experiments. DRB is a drug that specifically inhibits kinases that phosphorylate Serine 2 of the RNAPII C-terminal domain (CTD), thereby exclusively inhibiting transcriptional elongation [23]–[25]. RNA FISH analysis of primary transcripts demonstrated that inhibition of transcription with DRB or α-amanitin was indeed very efficient, with the percentage of cells showing active globin transcription going down from >70% in untreated cells to ∼3% and 0% in DRB- and α-amanitin-treated cells, respectively (Figure 1B). A western blot using an antibody detecting total RNAPII demonstrated that the high molecular weight band, which represents the active phosphorylated form of RNAPII is indeed almost absent upon α-amanitin treatment (Figure 1C). This was confirmed when we used an antibody specific for the actively elongating RNAPII, which is phosphorylated at the Serine 2 position of its CTD (Figure 1C). Accordingly, foci of Ser2 phosphorylated RNAPII, which define the nuclear sites of active transcription, disappeared upon transcription inhibition (Figure S1).

Chromatin immunoprecipitation (ChIP) experiments using an antibody against RNAPII demonstrated that treatment of erythroid cells with DRB results in reduced RNAPII occupancy at intron2 and exon3 of the β-major gene while the β-major promoter remains bound (Figure 1D), in agreement with DRB causing abortive transcription elongation [23]–[25]. In contrast, treatment with α-amanitin resulted in a strong reduction of RNAPII occupancy at all sites tested. RNAPII was no longer bound within the β-major gene and was almost absent from the hypersensitive sites in the LCR. Some RNAPII remained bound to the β-major promoter and the promoters of *Rad23A* and several other genes present at a gene-dense region of mouse chromosome 8, but in each case binding was strongly reduced (Figure 1D,E). These observations are in agreement with α-amanitin being an inhibitor of both transcription initiation and elongation by RNAPII [26]–[28].

We conclude that under our experimental conditions transcription is efficiently inhibited. As reported previously, DRB specifically reduces the elongating form of RNAPII while α-amanitin inhibits both the initiating and elongation form. Importantly, upon treatment with α-amanitin the active, Ser2 phosphorylated, form of RNAPII disappears almost completely and association of RNAPII with regulatory elements of the β-globin locus and other loci is lost or strongly reduced.

### LCR-gene interactions in the β-globin locus are not dependent on ongoing transcription

**Figure 2:**
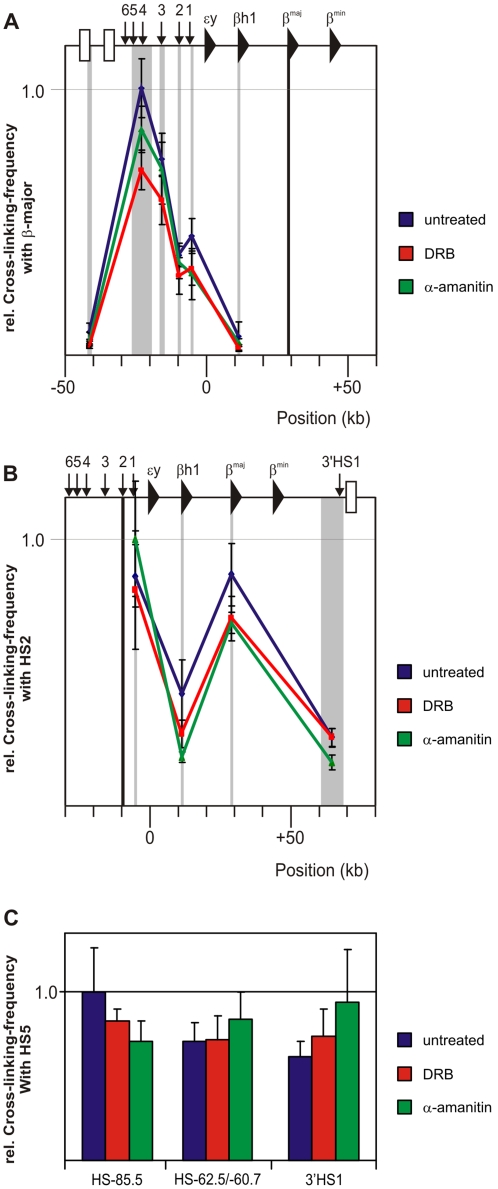
LCR-gene interactions in the β-globin locus are not dependent on ongoing transcription. (A–B) Locus wide cross-linking frequencies observed in untreated fetal liver cells (blue) DRB treated fetal liver cells (red) and α-amanitin treated fetal livers (green) are shown. The murine β-globin locus is depicted on top of each graph. X-axis shows position in the locus. Black shading shows the position and size of the ‘fixed’ HindIII fragment. Grey shading indicates position and size of other HindIII fragments analyzed. Standard-error-of-mean is indicated. Cross-linking frequencies are normalized to the highest interaction within an experiment and give an arbitrary value of 1. (A) Cross-linking frequencies for a restriction fragment containing the β-globin promoter. High crosslinking frequencies with restriction fragments containing the hypersensitive sites of the LCR are observed in all samples, indicating close proximity between the β-globin promoter and LCR (B) Cross-linking frequencies for a restriction fragment containing HS2 of the β-globin LCR. High crosslinking frequencies with the restriction fragment containing the β-globin promoter is observed in all samples, indicating close proximity between HS2 of the LCR and the β-globin promoter. (C) Bar graphs of cross-linking frequencies for a restriction fragment containing the CTCF-binding HS4/5 of the β-globin LCR with other selected CTCF-binding sites within the β-globin locus (i.e. HS-85.5, HS-62.5/-60.7 and 3′HS1). Blue bars depict untreated samples, red bars DRB treated samples and green bars α-amanitin treated samples. Cross-linking frequencies are normalized to the highest interaction within the experiment and give an arbitrary value of 1. Error bars indicate standard error of mean.

We next investigated if transcription inhibition had an effect on DNA contacts formed between regulatory sites in gene loci. The β-globin locus adopts an erythroid-specific spatial organization, the Active Chromatin Hub (ACH), in which the LCR and additional regulatory DNA elements spatially cluster with the actively transcribed β-globin genes [4]. We applied an improved version of 3C, 3C-qPCR [8], [29] to compare the conformation of the β-globin locus between E14.5 fetal liver cells cultured without or with either DRB or α-amanitin. The data showed that the hypersensitive sites of the LCR remain in close proximity to the β-major gene after transcription inhibition by DRB and α-amanitin (Figure 2A). This observation was confirmed when we analyzed cross-linking frequencies of a restriction fragment containing HS2, the classical enhancer of the LCR (Figure 2B). Like the LCR-promoter interactions, interactions between *cis*-regulatory elements HS-85.5, HS-60.7/62.5, 3′HS1 and HS5 of the LCR, that interact in a CTCF dependent manner [8], were unchanged after transcription inhibition (Figure 2C). The function of these elements is unknown as their presence or interaction is dispensable for high-level β-globin transcription [8], [30].

The finding that no considerable changes in interaction frequencies between these sites occurred while RNAPII binding to these sites was strongly reduced or even almost completely absent demonstrates that the maintenance of DNA loops formed between regulatory sites and genes within the β-globin locus does not depend on chromatin-bound RNAPII or ongoing transcription.

### The tissue specific β-globin locus does not switch its nuclear environment upon transcription inhibition

Using novel 4C technology, which allows for an unbiased genome-wide search for DNA loci that contact a given locus in the nuclear space, we recently demonstrated that the active β-globin locus in fetal liver contacts transcribed loci whereas the inactive β-globin locus in fetal brain contacts transcriptional silent loci elsewhere on the chromosome [15]. In contrast, the nuclear environment of a housekeeping gene present in a gene-dense region on chromosome 8 is very similar between the two tissues and consists of actively transcribed loci. This data suggests that the local nuclear environment of a locus is dependent on its transcriptional status.

We performed 4C analysis on control and α-amanitin treated E14.5 fetal liver cells to investigate the dependence of the β-globin nuclear environment on ongoing RNAPII transcription. Tailored microarrays containing 400.000 probes that each analyze a different DNA interaction and cover 7 complete mouse chromosomes were used [15]. Data were analyzed as described previously[15], using running mean algorithms with a window size of approximately 60 kb on true and randomly shuffled datasets to identify genome regions that show a significant proportion of interacting DNA fragments. These clusters of interacting DNA fragments are highly reproducible between biological replicates and represent truly interacting DNA regions as shown by many cryo-FISH experiments [15].

**Figure 3:**
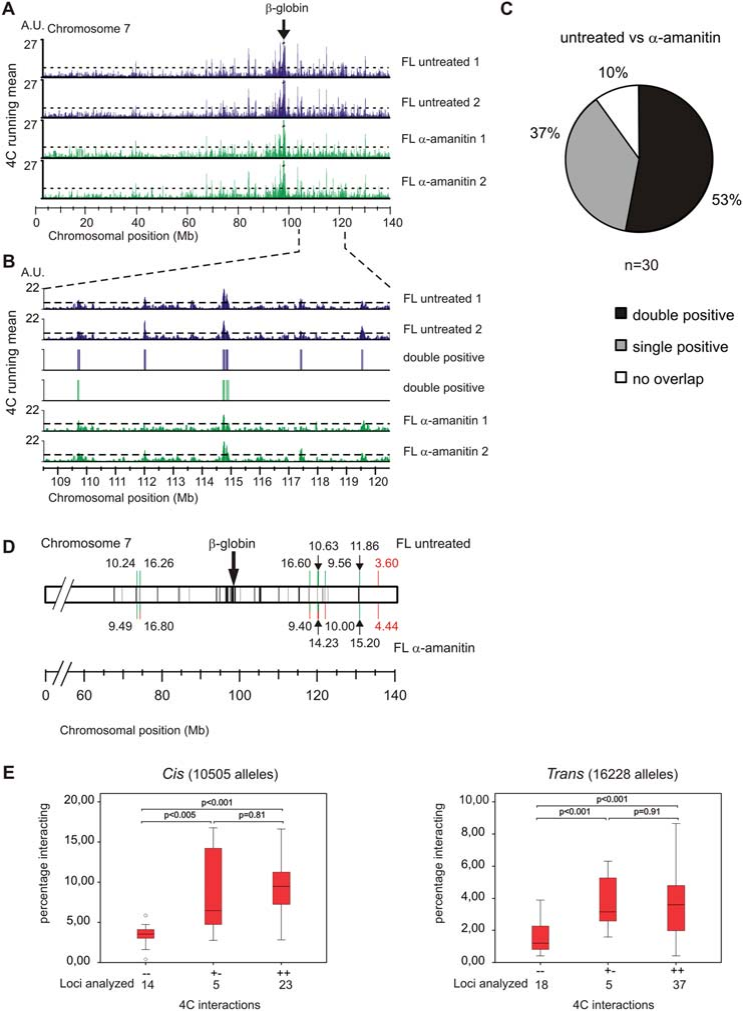
The tissue specific β-globin locus does not switch its nuclear environment upon transcription inhibition. (A) Running mean data for two untreated fetal liver (blue) and α-amanitin treated fetal liver (green) samples are shown. Peaks of interaction of the β-globin promoter that are above the threshold (dashed line) with several regions on chromosome 7 are observed. (B) Zoom in of the running mean data plotted along a 12-Mb region centered around 114.5 MB on chromosome 7. False discovery rate was set at 5% (dashed line). Highly reproducible clusters of interactions with the β-globin locus are observed in the two untreated fetal liver samples and the two α-amanitin treated fetal liver samples. Vertical bars indicate interactions that reach threshold levels in both duplicates. Chromosomal positions were based on National Center for Biotechnology (NCBI) build m34. (C) Indicated are the percentages of interacting regions on chromosome 7 observed for the β-globin locus in untreated fetal liver that are identified in both, a single or none of the α-amanitin treated fetal liver samples. (D) Schematic representation of cryo-FISH results. Grey shading indicates the regions identified (positive in both replicates) in untreated fetal livers, black shading indicates the regions identified (positive in both replicates) in α-amanitin treated fetal liver cells. Percentages of interaction with β-globin are indicated above the chromosome for untreated fetal liver cells or below the chromosome for α-amanitin treated fetal liver cells (black numbers indicate interacting, red non-interacting). Green bars indicate regions that are identified to be positive in both replicates by 4C technology. Red bars indicate regions identified to be negative in both replicates. Regions that are identified to be positive in only one replicate are indicated by a combined red and green bar. (E) Regions that score single positive after treatment while being double positive in untreated samples represent genuine interacting regions. Box plots representing our collective cryo-FISH data obtained for negative, single positive and double positive 4C regions for 102 different loci in different cell types scoring a total of 26733 alleles. Left panel depicts data obtained for interactions in *Cis*, right panel depicts data obtained for interactions in *Trans*. Horizontal bars represent the 10^th^, 25^th^, 50^th^ (median), 75^th^ and 90^th^ percentiles, and *p* values for pairs of samples are indicated. The *p* value for a pair of samples was determined by an independent samples t-test for equality of means. Circles represent single values identified as outliers.

Replicate experiments that analyze interactions with the β-major promoter with and without transcription are depicted in Figure 3A. When we compared the data for the silent β-globin locus in fetal liver cells treated with α-amanitin with the data we previously obtained for the silent β-globin locus in fetal brain [15], we found that they differed completely (τ = 0.028 on average; Spearman's rank correlation). This means that the β-globin locus in fetal liver, which is silenced through treatment by α-amanitin displays long-range interactions, which differ from the long-range interactions observed for the developmentally silenced β-globin locus in fetal brain. Importantly, comparison of β-globin interactions observed in α-amanitin treated versus untreated samples demonstrated that these are highly correlated (τ = 0.66 on average; Spearman's rank correlation). Moreover, a single hybridization of a DRB treated fetal liver sample correlated similarly to the untreated cultured fetal liver cells (τ = 0.68 on average; Spearman's rank correlation). These correlations are comparable to the correlation observed between replicates of the untreated samples (τ = 0.70; Spearman's rank correlation; Table S1), suggesting that drug-induced silencing of transcription has minor impact on DNA contacts formed by the β-globin locus.

In order to further characterize the 4C data, we defined positive regions of interaction using a threshold value that allows a false discovery rate of 5% (Figure 3A). The threshold used was based on the running mean distribution of the data after it was randomly shuffled, which reveals to what extent clustering of positive hybridization signals can occur by chance. Genomic regions were defined as interacting if they met this threshold in two independent replicate experiments (i.e. double positive regions)[15].

Using these criteria we identified 30 interacting regions in the untreated sample and most were the same regions, containing actively transcribed genes, as previously observed in freshly dissected, uncultured fetal liver cells [15]. Of these 30 regions, 16 (53%) were also found to be double positive in α-amanitin treated samples (Figure 3A,B).

We validated these interactions using cryo-FISH. This technique has the advantage over three-dimensional FISH in that it better preserves the nuclear ultrastructure and offers improved resolution in the z-axis by the preparation of ultrathin cryosections [22]. Routinely, 250 loci or more were analyzed per cryo-FISH experiment and a person not aware of the two loci under investigation determined how frequently their hybridization signals overlapped. When we applied cryo-FISH to a region that scored double negative in the 4C analysis for the untreated and α-amanitin treated samples we found an interaction frequency of ∼4% between these loci, which is similar to the background interaction that was found previously [15]. In contrast, the regions we tested and that scored to be interacting based on the 4C data indeed had interaction frequencies significantly above the background (>9%; p<0.001, G-test), both in untreated and α-amanitin treated cells. Surprisingly, when we applied cryo-FISH to four regions that scored double positive in untreated samples but not in α-amanitin treated samples we found interaction frequencies significantly above background (>9%; p<0.001, G-test) not only in untreated cells but also in the α-amanitin treated cells (Figure 3D). This observation shows that these regions are still in close proximity in the α-amanitin treated samples and demonstrates they are scored false negative by 4C technology.

We therefore examined more closely the 4C data of our α-amanitin treated samples, in particular the 14 out of 30 regions (47%) that were no longer identified as interacting in these samples. We noticed that they often (11/14) scored positive for interaction in one of the 4C experiments (Figure S2), while they just failed to reach the threshold in the replicate experiment (i.e. single positive regions) (Figure 3B,C and Figure S2). This suggests that these regions were not identified by 4C as interacting due to the stringent threshold we applied.

To find out whether 4C more often scores such regions as false negative we collected all our cryo-FISH data also from other ongoing projects. Cryo-FISH analysis of 104 different intra and interchromosomal interactions showed that regions, which scored double positive by 4C, interact significantly more frequently than 4C-negative regions (p<0.001, independent samples T-test). Importantly, this was also true for the 10 regions tested that scored single positive by 4C (but double positive under a different condition) (Figure 3E). Thus, counting by cryo-FISH of more than 26,000 alleles representing 104 different interactions confirmed that 4C very accurately identifies long-range DNA interactions. The data also demonstrate that regions identified as 4C-positive in only one of the two experiments in general represent true, i.e. non-random, interactions, at least when they are also scored double positive by 4C under a different experimental condition. Therefore, these single positives are regions are not identified as interacting by 4C, using our stringent criteria, because they fail to reach the threshold in one of the two 4C replicate experiments. Yet, they often do interact, as determined by cryo-FISH. In general, these are long-range interactions that occur in less than 5% of the cells. Their identification by 4C relies on the detection of very rare ligation products that can easily be missed if the experiment is carried out under sub-optimal conditions.

We conclude that the great majority of interactions (at least 90%) between the β-globin locus and other active gene loci are independent of ongoing transcription. This percentage may even be higher, as the 1 out 3 regions that we tested in cryo-FISH and which scored negative in each 4C replicate of the α-amanitin treated samples nevertheless also appeared to interact frequently (10%) with the β-globin locus (Figure 3D and Table S2). Thus, none of the in total 7 regions tested was found to loose interaction when transcription was blocked.

Previously we demonstrated that the β-globin locus switches its nuclear environment in relation to its activity when this activity is governed by its developmental status [15]. Here we demonstrate that once established, the long-range interactions of the active β-globin locus with other active genes are not dependent on the process of ongoing transcription or on the binding of RNAPII to regulatory elements.

### The housekeeping gene *Rad23a* does not switch its nuclear environment upon transcription inhibition

**Figure 4:**
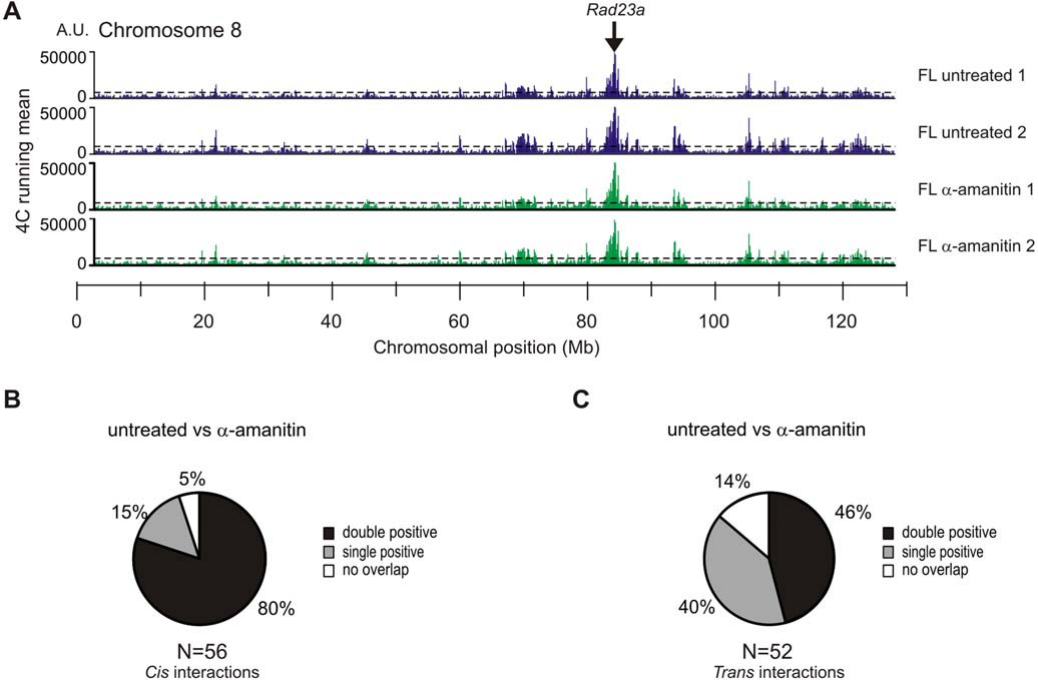
The housekeeping gene *Rad23a* does not switch its nuclear environment upon transcription inhibition. (A) Running mean data for two untreated fetal liver (blue) and α-amanitin treated fetal liver (green) samples is shown. Peaks of interaction of the *Rad23a* promoter above the threshold (dashed line) with several regions on chromosome 8 can be observed. False discovery rate was set at 5% (dashed line). Chromosomal positions were based on NCBI build m34. (B) Indicated are the percentages of interacting regions observed for *Rad23a* in *cis* on chromosome 8 in untreated fetal liver that are identified in both, a single or none of the α-amanitin treated fetal liver samples. (C) Indicated is the percentages of *trans*-interacting regions observed for *Rad23a* in untreated fetal liver that are identified in both, a single or none of the α-amanitin treated fetal liver samples.

The β-globin locus is expressed at an exceptionally high rate, in a tissue specific manner and therefore it is possible that the conserved nuclear environment of the β-globin locus after transcription inhibition is specific for this locus. To analyze the generality of this observation we investigated the nuclear environment of *Rad23a*, a housekeeping gene that resides in a gene-dense cluster of mostly housekeeping genes on chromosome 8 that is active in E14.5 fetal liver as well as in brain [15]. As was found for the β-globin locus, the great majority of long-range interactions in *cis* (and in *trans*) of the Rad23a locus remain the same between uncultured and cultured fetal liver cells and was made with regions of high transcriptional activity. A comparison between 4C data obtained with the untreated and α-amanitin treated samples showed that these are highly correlated (Figure 4A)(τ = 0.8 on average; Spearman's rank correlation). The correlation with a single DRB treated sample was found to be even higher (τ = 0.94 on average; Spearman's rank correlation).

As for the β-globin locus we analyzed the 4C data and identified and compared interacting regions. Forty-four out of fifty-six (80%) of the double positive regions found on chromosome 8 in the untreated fetal liver cells were conserved in α-amanitin treated fetal liver cells while another 8/56 regions (15%) were found in one of the two replicate α-amanitin treated samples (Figure 4B). Thus, the great majority of long-range interactions with other gene loci elsewhere on the same chromosome were maintained in the absence of ongoing transcription.

The mouse β-globin locus resides within its own chromosome territory [31] and as a consequence does not display any interchromosomal contacts with loci located on different chromosomes [15]. In contrast, the gene-dense region containing *Rad23a* resides mostly on the edge or outside of its chromosome territory [32] and in agreement it was previously found to interact with regions on other chromosomes [15]. We analyzed whether these inter-chromosomal interactions depended on ongoing transcription by investigating interactions with six unrelated chromosomes (7, 10, 11, 12, 14 and 15) that were represented on the array. Using a running median algorithm with a false discovery rate of 0% to determine threshold values, we found 54 regions in *trans* that interact with the Rad23a locus. Twenty-four (46%) of these inter-chromosomal *Rad23a* interactions were conserved between untreated and α-amanitin treated fetal liver cells (Figure 4C), while another 21 (40%) of the inter-chromosomal *Rad23a* interactions identified in untreated cells were found to be positive in one of the replicates of the α-amanitin treated fetal liver cells.

We verified the 4C data by measuring co-localization frequencies of *Rad23a* alleles with selected chromosomal regions in *trans* using cryo-FISH (Table S3). Inter-chromosomal regions that associate with the Rad23a locus based on the 4C data indeed all have co-localization frequencies (2.7%–8.3%) above the inter-chromosomal background level (0%–1.96%; *P*<0.05 *G* test). The two regions tested that scored positive for interaction in only one 4C dataset do also interact with *Rad23a* in α-amanitin treated cells, demonstrating again that such single positive regions also represent true associations.

These observations demonstrate that the long-range interactions, in *cis* as well as in *trans*, of the ubiquitously expressed Rad23a locus with other active genes are also not dependent on the process of ongoing transcription or on the binding of RNAPII to regulatory elements.

### The chromatin fiber of transcriptionally inhibited gene loci remains in an active state

**Figure 5:**
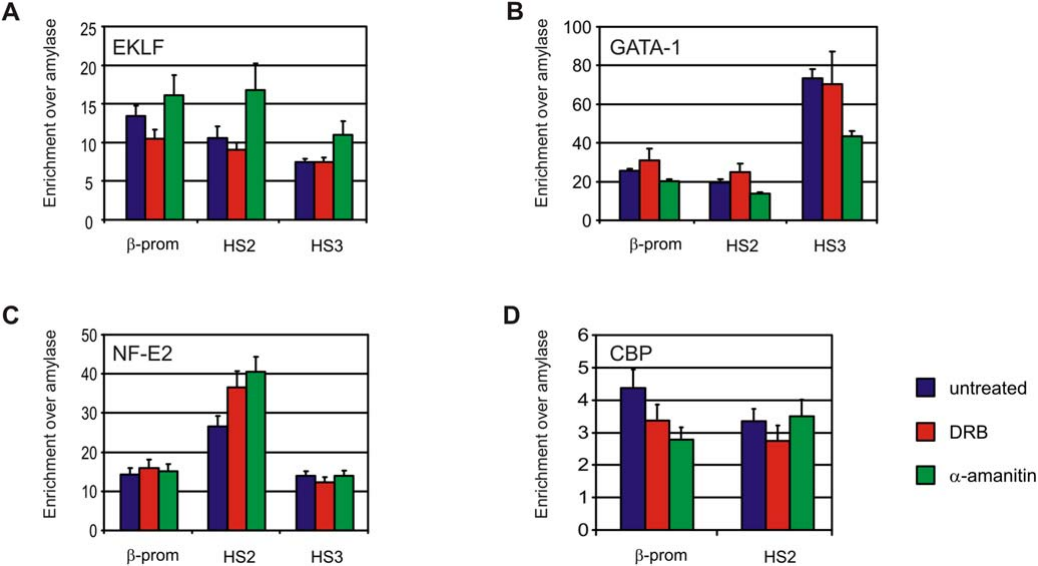
Key erythroid transcription factors remain bound to regulatory sites of the β-globin locus. Binding of (A) EKLF, (B) GATA-1, (C) NF-E2 and (D) CBP at β-globin regulatory elements. Blue bars depict untreated fetal liver samples, red bars DRB treated fetal liver samples and green bars α-amanitin treated fetal liver samples. Enrichment is relative to amylase. Error bars indicate standard error of mean.

**Figure 6:**
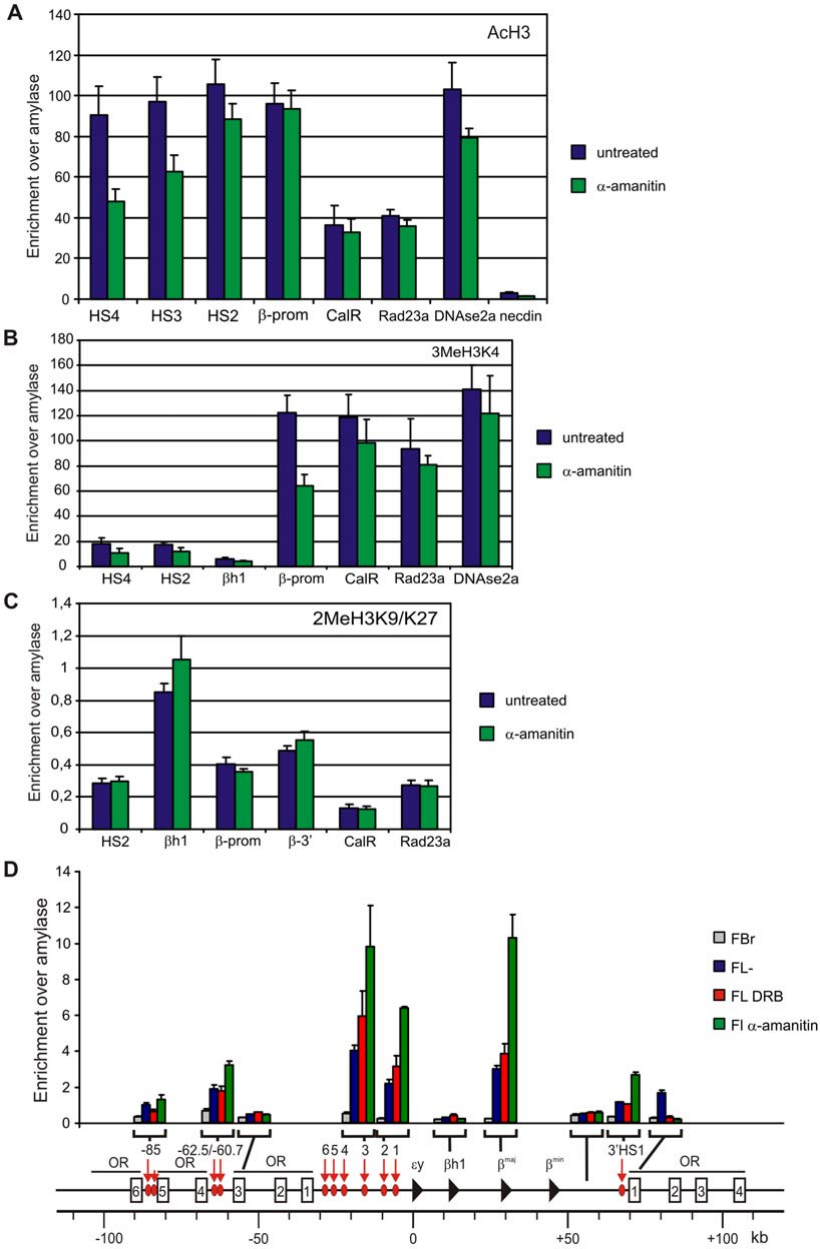
The chromatin of loci remains in an active state after transcription inhibition. (A) Acetylation of histone H3 at regulatory sites of the β-globin and Rad23a locus (B) dimethylation of lysine 9 and 27 of histone H3 at regulatory sites of the β-globin and Rad23a locus (C) Trimethylation of lysine 4 of histone H3 at regulatory sites of the β-globin and Rad23a locus. (D) Detection of histone depleted chromatin at regulatory elements of the β-globin locus using FAIRE. Enrichment is relative to amylase. Grey bars depict fetal brain samples, blue bars depict untreated fetal liver samples, red bars depict DRB treated fetal liver samples and green bars α-amanitin treated fetal liver samples. Error bars indicate standard error of mean.

We set out to search for other properties that may underlie the architecture of chromatin in the nucleus. Active and inactive chromatin domains separate in the nucleus [15], [33] and are characterized by a distinct organization of the chromatin fiber. We therefore investigated to what extent the inhibition of transcription influences the status of the chromatin fiber at gene loci. We first analyzed binding of relevant *trans*-acting factors to the β-globin locus. EKLF, GATA-1 and NF-E2, three erythroid-specific transcription factors implicated in β-globin gene regulation and chromatin modification of the β-globin locus, remain bound to HS3, HS2 and the β-major promoter after transcription inhibition, with only minor changes in binding efficiencies (Figure 5A–D). Interestingly, two of these factors, EKLF and GATA-1, were previously shown to directly mediate the formation of LCR-gene contacts in the β-globin locus [6], [7], [34]. Binding of the co-activator CBP, a histone acetyl transferase that has also been suggested to function in long-range β-globin gene activation [35], was also nearly the same upon transcription inhibtion (Figure 5D). In agreement, acetylated histone H3 levels (Figure 6A) and tri-methylation of lysine 4 at histone H3 (Figure 6B) remained unaltered upon α-amanitin treatment at most regulatory sites of the β-globin and Rad23a locus. Although a reduction in enrichment for these active chromatin marks was observed at some regulatory sites of the β-globin locus they were considerably more enriched than inactive loci such as Necdin, βh1 and Amylase. Di-methylation of lysine 9 and 27 of histone H3, a chromatin mark that is associated with inactive genes, also remained unaltered upon α-amanitin treatment (Figure 6C).

The continued binding of transcription factors suggests that regulatory sites are still formed in the absence of transcription. To investigate this we used Formaldehyde Assisted Identification of Regulatory Elements (FAIRE) [36]. In FAIRE, chromatin is crosslinked with formaldehyde in vivo, sheared by sonication, and phenol-chloroform extracted. The DNA recovered in the aqueous phase is enriched for nucleosome-depleted DNA, which is coincident with the location of DNaseI hypersensitive sites, transcriptional start sites and active promoters. All regulatory elements tested in the β-globin locus were readily identified in both untreated and transcriptionally inhibited fetal liver cells. They were absent from fetal brain cells, in line with them being erythroid-specific hypersensitive sites (Figure 6D). Regulatory elements of the Rad23a locus were also readily detected by FAIRE in untreated and α-amanitin treated fetal liver cells (Figure S3). Some of the regulatory sites detected by FAIRE were more prominently present in the α-amanitin treated sample as compared to untreated or DRB treated cells (Figure 6D). The regulatory elements identified by FAIRE contained less histone H3 (Figure S4) [37] but this did not decrease any further upon transcription inhibition with α-amanitin. We suggest that the observed decrease in crosslinkability of these regulatory sites upon α-amanitin treatment reflects the loss of RNAPII binding that was observed at these sites (Figure 1D).

Taken together, our results demonstrate that although binding of RNAPII to the locus is severely reduced upon transcription inhibition, the association of key transcription factors with the regulatory sites of the β-globin locus does not change or is only mildly affected. Accordingly, in the absence of transcription the chromatin fiber remains in an active state as is demonstrated by the observation that the regulatory elements of the β-globin locus as well as the Rad23a locus retain their active chromatin marks and maintain an open chromatin configuration.

## Discussion

It is often thought that transcription, or the nuclear distribution of RNAPII, plays an important role in shaping the genome in the interior of the nucleus [16], [17], [38], [39]. Based on these ideas it was predicted that inhibition of transcription changes DNA folding and eliminates looping [18], [20]. Several microscopy studies have addressed the role of transcription in genome organization with contradicting results (e.g. [22], [33], [40], [41]). The recent development of 3C and 4C technology has allowed us to carry out a detailed high-resolution study to test the role of transcription in genome organization.

We inhibited transcription in fetal liver cells with DRB and α-amanitin, using stringent conditions [21], [22], [28]. We observed a block in transcription, an almost complete absence of ser2 phosphorylated RNAPII, and a strong reduction of RNAPII occupancy at the β-major promoter and hypersensitive sites of the β-globin LCR (Figure 1B–D). Crucially, while the amount of chromatin-bound RNAPII is strongly reduced, interactions between regulatory elements within the β-globin locus are detected at normal frequencies by 3C analysis (Figure 2). In addition, based on unbiased high-throughput 4C analysis and high-resolution cryo-FISH (we scored interactions with 12 different loci, counting more than 5000 alleles), we find that there is no large-scale change in long-range intra- or inter-chromosomal interactions of the β-globin locus and Rad23a locus after inhibition of transcription by α-amanitin (Figure 3 and 4).

One could argue that residual RNAPII bound to chromatin is responsible for the maintenance of the long-range interactions observed. However, the amount of RNAPII that remains bound to the β-globin promoter or to the β-globin LCR is similar or lower than the amount found in un-induced primary erythroid progenitor cells where interactions between HS2 and the β-globin promoter are absent [34]. In fact, RNAPII binding at for example HS4 is reduced to background levels, but interaction frequencies of HS4 with the active β-globin gene are unaltered in α-amanitin treated cells (Figure 1D, 2A). More generally speaking, ChIP, 3C and 4C all measure steady state levels in a cell population and a severe drop in the levels of chromatin-associated RNAPII as measured by ChIP is expected to result in reduced DNA:DNA interaction frequencies as measured by 3C and 4C, if such interactions were mediated by RNAPII. This is not what we observe.

The different phosphorylated versions of RNAPII have a different nuclear distribution, as uncovered by immunocytochemistry using specific antibodies [21]. The elongating, Ser-2 phosphorylated, version of RNAPII is the isoform that specifically accumulates at sites of active transcription [22], [42]. The foci that are formed are often referred to as transcription factories. The relevance of these foci is still unclear though, as they are not detected with fluorescently labeled RNAPII in living cells [43]. We readily detect these foci in fixed untreated cells, but they are almost completely absent from fixed cells that are treated with α-amanitin (Figure S1).

Taken together our observations strongly suggest that maintenance of long-range chromosomal interactions is not dependent on ongoing RNAPII transcription or chromatin-bound RNAPII, whether or not organized in transcription factories [16]–[18], [20], [44]. The data therefore argue against models that suggest that engaged RNA polymerases function as the ties of chromatin loops [13], [16], [17], [19], [44].

We cannot exclude that a first round of transcription, for example in early G1, is required for loci to adopt their favorite position in the nucleus and to establish contacts with other loci [45]. However, if such long-range contacts are dynamic just like the DNA interactions between *ci*s-regulatory elements within a locus [24], [46], they are expected to be disrupted during the time of α-amanitin treatment. If this is indeed the case they are apparently re-established in the absence of transcription, which argues against a role for transcription in this process.

We observed that upon α-amanitin treatment the locus remains in an epigenetic chromatin state associated with transcriptionally active regions. Erythroid specific transcription factors EKLF and GATA-1, both implicated in establishment of LCR-β-major promoter contact [6], [7], as well as NF-E2 remain bound to their cognate binding sites upon transcription inhibition (Figure 5A). Moreover, the histone acetyl transferase CBP, previously implicated in long-range β-globin activation [35] remains associated with the β-globin LCR and promoter (Figure 5A). This reinforces the idea that these (erythroid specific) transcription factors, probably in complex with other proteins, are mainly responsible for the stability of the DNA interactions between regulatory elements of the β-globin locus. The regulatory sites of the β-globin and Rad23a locus retain their active chromatin marks and their physical and chemical properties that allow their identification by FAIRE (Figure 5B–C and Figure S3).

Our analysis allows the appreciation of three states of the β-globin locus (Table 1). First, in fetal brain cells a developmental program transcriptionally inactivates the β-globin locus. In these cells the β-globin locus has an inactive chromatin state associated with transcriptionally inactive loci, with no RNAPII bound at regulatory regions. Here, the locus displays long-range chromatin interactions with other transcriptionally silent loci [15]. Second, in fetal liver cells the β-globin locus is transcriptionally highly active, accordingly has an active chromatin state associated with transcriptionally active regions and RNAPII is highly enriched at regulatory regions. Here, the locus interacts with other transcriptionally active loci [15]. Third, in α-amanitin treated fetal liver cells, the β-globin locus is transcriptionally silenced and RNAPII is largely evicted from the gene body and the regulatory regions. The locus retains its overall active chromatin profile though and it interacts with the same regions that are contacted by the highly transcribed β-globin locus (Table 1).

**Table 1:** Observed states of the β-globin locus

Cell type	Transcription^a^	RNAPII^b^	Chromatin state^c^	Long-range interactions^d^
Fetal Brain	−	−	inactive	inactive loci e
Fetal Liver	++	++	active	active loci e
Fetal Liver+α-amanitin	−	−/+	active	“active loci”

a Transcriptional activity of the β-globin locus.

b presence of RNAPII at regulatory sites of the β-globin locus as determined by ChIP.

c as judged by the presence of acetylated histone H3 at regulatory sites of the β-globin locus as determined by ChIP and/or nucleosome occupancy as determined by FAIRE.

d Activity of loci found to be interacting with the β-globin locus. “active loci” refers to the same subset of loci found to be active in fetal liver cells but now inactive due to α-amanitin treatment.

e see ref. [15] for details.

Previous studies suggested a role for chromatin modifications in large-scale movement of loci away from heterochromatic regions and the subsequent changes in transcription and replication timing in the human β-globin locus [47] and the CFTR locus [48]. Our results strengthen this notion and are consistent with a model in which the overall chromatin state, i.e. the organization and modification of nucleosomes along the DNA fiber and the binding of specific *trans*-acting factors, more than the process of transcription or the nuclear distribution of RNAPII, determines the nuclear positioning of DNA loci and their interactions with other genomic sites according to self-organizing principles [2], [49], [50].

## Materials and Methods

### Culture and drug treatment of fetal liver cells

Freshly dissected E14.5 fetal livers were re-suspended in 2 ml differentiation medium (StemPro-34™ containing 5 units/ml Epo and 1 mg/ml iron-saturated human transferrin). DRB was added to a final concentration of 100 µM and incubated for 3 h at 37°C/5%CO_2_. α-Amanitin was added to a final concentration of 100 µg/ml and incubated for 5 h at 37°C/5%CO_2_. Control cells were incubated for 5 h.

### RNA-FISH

RNA-FISH was done as described previously [51].

### Western Blotting

Whole cell lysates where boiled in SDS loading buffer and run on NuPAGE 4–12% precast Bis-Tris gels (Invitrogen). Proteins were transferred to nitrocellulose membrane and proteins were detected using the ECL western blotting detection reagent (RPN2106) from GE Healthcare. Antibodies: 1^st^ AB: pan RNAPII (N-20) sc-899 Santa-Cruz; Ser2 acetylated RNAPII (H5) ab-24758 Abcam. 2^nd^ AB: horseradish peroxidase (HRP)-labeled sheep anti-mouse IgG (NXA931) from Amersham Bioscience.

### Immuno staining of cells

Cells were spotted on poly-lysine coated slides (Sigma) and fixed in 3.7% formaldehyde/5% acetic acid and permeabilized using pepsin and incubated with antibodies as described before [51]. Antibodies used: 1^st^ AB: Ser2 acetylated RNAPII (H5) ab-24758 Abcam; 2^nd^ AB: Alexa Fluor 594 conjugated Rabbit anti-Mouse IgG from Invitrogen.

### Confocal microscopy

Image stacks (Z sections spaced ∼0.34 µm apart) were collected using a Zeiss LSM510Meta confocal microscope equipped with a 63× oil Plan-Apochromar n.a. 1.4 objective. The image stacks were projected onto a single plane using Volocity image analysis software (http://www.improvision.com/products/volocity) and analyzed in Photoshop CS (Adobe).

### Chromatin Immunoprecipitation (ChIP)

ChIP was performed as described in the Upstate protocol (http://www.upstate.com), except that some samples were cross-linked with 2% formaldehyde for 5 minutes at room temperature without creating differences in enrichment for the transcription factors tested. Quantitative real-time PCR (Opticon I, MJ Research) was performed using SYBR Green (Sigma) and Platinum Taq DNA Polymerase (Invitrogen), under the following cycling conditions: 94°C for 2 min, 44 cycles of 30 s at 94°C, 60 s at 55°C, 15 s at 72°C and 15 s at 75°C (during which measurements are taken). Enrichment was calculated relative to Amylase and values were normalized to input measurements. Antibodies used: RNAPII (N-20; sc-899), NF-E2 (C-19; sc-291), GATA-1 (N6; sc-265) and CBP (A22; sc-369) from Santa Cruz Biotechnology, Ac-H3 (#06-599) and anti-tri-methyl Histone H3 K4 (#07-473) from Upstate, PanH3 (#ab1791) and anti-di-methyl Histone H3 K9/K27 (#ab7312) from Abcam. Anti-EKLF (5-V; in-house generated) was kindly provided by S. Phillipsen. Primer sequences are available on request.

### 3C Analysis

3C analysis was performed essentially as described [8], [29] using HindIII as the restriction enzyme. Quantitative real-time PCR (Opticon I, MJ Research) was performed with Platinum Taq DNA Polymerase (Invitrogen) and double-dye oligonucleotides (5′FAM+3′TAMRA) as probes, using the following cycling conditions: 94°C for 2 min and 44 cycles of 15 s at 94°C and 90 s at 60°C. Primer sequences are available on request.

### 4C Analysis

4C was performed as described before [15]. Raw data is available in the Gene Expression Omnibus (GEO) database under accession number GSE10170. 4C data analysis and Array design were performed as described before except that hybridization with differentially labeled genomic DNA was omitted. Each array was hybridized with two independently processed experimental samples labeled with alternate dye orientations. A running mean algorithm was performed for analysis of the *cis*-interactions directly on uncorrected signal intensities giving similar results as after using the signal ratio 4C-sample/genomic DNA. Results shown are based on a window size of 29 probes (on average 60 kb) and were compared with the running mean performed across randomized data. This was done for each array separately. Consequently, all measurements were judged relative to the amplitude and noise of that specific array. We determined the correlation between long-range interactions of the β-globin locus in cultured fetal liver cells and previous data obtained for HS2 in freshly dissected fetal liver cells [15]. Correlation was high (τ≅0.5; Spearman's rank correlation) demonstrating that culturing of erythroid cells does not drastically influence the interactions of the β-globin locus with distal regions. The threshold level was determined using a top-down approach to establish the minimal value for which the false discovery rate (FDR) <0.05.

Next, duplicate experiments were compared. Windows that met the threshold in both duplicates were considered positive. When comparing randomized data, no windows were above threshold in both duplicates. Positive windows directly adjacent on the chromosome template were joined (no gaps allowed), creating positive areas.

In defining interacting regions in *trans*, we took a similar approach again omitting hybridization with differentially labeled genomic DNA. As described before [15] we applied a running median, using a window size of 29 probes, directly on uncorrected signal intensities giving similar results as after using the signal ratio 4C-sample/genomic DNA. The threshold was set at an FDR of 0%. Thus, a region was called interacting when, in both duplicates the median signal was higher than any signal found in the respective randomized data sets.

### Cryo-FISH

Cryo-FISH was performed as described before [22] with minor adaptations. Briefly, After culturing and drug treatment, E14.5 fetal liver single cell suspensions were fixed for 20 min in 4% paraformaldehyde/250 mM HEPES (pH 7.5), followed by another fixation step of 2 h in 8% paraformaldehyde at 4°C. Consecutively the single cell suspension was briefly centrifuged and embedded in 10% gelatin. Fixed tissue blocks were immersed in 2.3 M sucrose for 20 min at room temperature (18–24°C), mounted on a specimen holder and snap-frozen in liquid nitrogen. Tissue blocks were stored in liquid nitrogen until sectioning. Ultrathin cryosections of approximately 200 nm were cut using a Reichert Ultramicrotome E equipped with a cryo-attachment (Leica). For hybridization, sections were washed with PBS to remove sucrose, treated with 250 ng/ml RNase in 2× SSC for 1 h at 37°C, incubated for 10 min in 0.1 M HCl, dehydrated in a series of ethanol washes and denatured for 8 min at 80°C in 70% formamide/2× SSC, pH 7.5. Sections were again dehydrated directly before probe hybridization. We co precipitated 500 ng labeled probe with 5 µg of mouse Cot1 DNA (Invitrogen) and dissolved it in hybridization mix (50% formamide, 10% dextran sulfate, 2× SSC, 50 mM phosphate buffer, pH 7.5). Probes were denatured for 5 min at 95°C, reannealed for 30 min at 37°C and hybridized for at least 40 h at 37°C. After post hybridization washes, nuclei were counterstained with 20 ng/ml DAPI (Sigma) in PBS/0.05% Tween-20 and mounted in Prolong Gold anti fade reagent (Molecular Probes).

Images were collected with a Zeiss Axio Imager Z1 epifluorescence microscope (100× plan apochromat, 1.4× oil objective), equipped with a charge-coupled device (CCD) camera and Isis FISH Imaging System software (Metasystems). In most cases a minimum of 250 β-globin or *Rad23a* alleles were analyzed and scored (by a person not knowing the probe combination applied to the sections) as overlapping or nonoverlapping with BACs located elsewhere in the genome. In 3 cases a more limited number of β-globin alleles (>123) was counted. Replicated goodness-of-fit tests (*G* statistic) were performed to assess significance of differences between values measured for 4C-positive versus 4C-negative regions.

### FISH probes

The following BAC clones (BACPAC Resources Centre) were used: RP23-370E12 for β-globin, RP24-332F3 for chromosome 7 at 73.9 Mb, RP23-455N12 for chromosome 7 at 121.45 Mb, RP23-410I10 for chromosome 7 at 86.7 Mb, RP24-263G24 for chromosome 7 at 119.5 Mb, RP24-142M15 for chromosome 7 at 117.4 Mb, RP23-143F10 for chromosome 7 at 130.1 Mb, RP23-470N5 for chromosome 7 at 73.1 Mb, RP23-269O10 for chromosome 7 at 134.8 Mb (olfactory receptor gene cluster), RP24-136A15 for *Rad23a*, RP23-32C19 for chromosome 7 at 118.3 Mb, RP24-334N22 for chromosome 7 at 84.1 Mb, RP24-199K7 for chromosome 11 at 119.8 Mb, RP24-534F13 for chromosome 15 at 76.6 Mb, RP23-311P1 for chromosome 11 at 102.2 Mb.

Random primer–labeled probes were prepared using BioPrime Array CGH Genomic Labeling System (Invitrogen). Before labeling, DNA was digested with *Dpn*II and purified with a DNA Clean-up and Concentrator 5 Kit (Zymo Research). Digested DNA (300 ng) was labeled with SpectrumGreen dUTP (Vysis) or Alexa Fluor 594 dUTP (Molecular Probes) and purified through a GFX PCR DNA and Gel Band Purification kit (Amersham Biosciences) to remove unincorporated nucleotides. The specificity of labeled probes was tested on metaphase spreads prepared from mouse embryonic stem (ES) cells.

### Formaldehyde Assisted Identification of Regulatory Elements (FAIRE)

Faire was performed as described before [36] except that selected genomic sites were analyzed by real-time Q-PCR. Primer sequences are available on request.

## Supporting Information

Table S1 Tabel showing spearman rank after quantile normalisation and running mean (window 29 probes) across data with 2 Mb around globin locus deleted (96–100 Mb)
(0.03 MB DOC)

Table S2 Overview of cryo-FISH results in cis for beta-globin
(0.04 MB DOC)

Table S3 Overview of cryo-FISH results in trans for Rad23a
(0.03 MB DOC)

Figure S1 Foci of the actively elongating form of RNAPII (RNAPII transcription factories) are absent after α-amanitin treatment of fetal liver cells
(0.29 MB DOC)

Figure S2 Clusters of *cis*-interactions for the β-globin locus
(0.76 MB DOC)

Figure S3 Active regulatory sites within the Rad23a locus remain in an active chromatin state after transcription inhibition while inactive regions remain inactive.
(0.26 MB DOC)

Figure S4 Nucleosome occupancy does not change upon transcription inhibition.
(0.21 MB DOC)
