# Supplementary material for: Maintenance of Long-Range DNA Interactions after Inhibition of Ongoing RNA Polymerase II Transcription
Source: PLoS One. 2008 Feb 20;3(2):e1661. doi: 10.1371/journal.pone.0001661 (PMC2243019; doi:10.1371/journal.pone.0001661)
Supplement: Table S1 — Tabel showing spearman rank after quantile normalisation and running mean (window 29 probes) across data with 2 Mb around globin locus deleted (96–100 Mb) (0.03 MB DOC) [file pone.0001661.s001.doc]

# Table S1.

spearman rank after quantile normalisation and running mean (window 29 probes) across data with

2 Mb around globin locus deleted (96-100 Mb)

|  | ut 1 | ut 2 | DRB | alpha 1 | alpha 2 | FL 1 | FL 2 | BR 1 | BR 2 |
| --- | --- | --- | --- | --- | --- | --- | --- | --- | --- |
| ut 1 | x |  |  |  |  |  |  |  |  |
| ut 2 | 0.702 | x |  |  |  |  |  |  |  |
| DRB | 0.706 | 0.638 | x |  |  |  |  |  |  |
| alpha 1 | 0.592 | 0.617 | 0.644 | x |  |  |  |  |  |
| alpha 2 | 0.762 | 0.657 | 0.668 | 0.585 | x |  |  |  |  |
| FL 1 | 0.481 | 0.515 | 0.392 | 0.386 | n.d. | x |  |  |  |
| FL 2 | 0.475 | 0.492 | 0.373 | 0.371 | n.d. | 0.654 | x |  |  |
| BR 1 | -0.012 | -0.035 | 0.037 | 0.032 | n.d. | 0.033 | 0.263 | x |  |
| BR 2 | 0.006 | -0.012 | 0.073 | 0.024 | n.d. | 0.080 | 0.228 | 0.611 | x |

ut = β-major data for untreated & cultured fetal liver

DRB = β-major data for DRB treated & cultured fetal liver

alpha = β-major data for alpha-amanitin treated & cultured fetal liver

FL = HS2 data for uncultured fetal liver

BR = HS2 data for uncultured fetal brain
