# Supplementary material for: Maintenance of Long-Range DNA Interactions after Inhibition of Ongoing RNA Polymerase II Transcription
Source: PLoS One. 2008 Feb 20;3(2):e1661. doi: 10.1371/journal.pone.0001661 (PMC2243019; doi:10.1371/journal.pone.0001661)
Supplement: Table S2 — Overview of cryo-FISH results in cis for beta-globin (0.04 MB DOC) [file pone.0001661.s002.doc]

# Table S2. Overview of cryo-FISH results *in cis* β-globin

***Cis*** interactions

| BACs | Loci | **Treatment** | **4C** | **Overlapping** | **Apart** | **N** | **% overlap** | G-test |
| --- | --- | --- | --- | --- | --- | --- | --- | --- |
| E12 x 269O10 | β-globin x 134.8 mb chr.7 OR | untreated | -- | 9 | 241 | 250 | 3.60 |  |
| E12 x 269O10 | β-globin x 134.8 mb chr.7 OR | α-amanitin | -- | 10 | 215 | 225 | 4.44 |  |
| E12 x 470N5 | β-globin x 73.1 mb chr.7 | untreated | ++ | 26 | 228 | 254 | 10.24 | P<0.001 |
| E12 x 470N5 | β-globin x 73.1 mb chr.7 | α-amanitin | ++ | 24 | 229 | 253 | 9.49 | P<0.001 |
| E12 x 143F10 | β-globin x 130 mb chr.7 | untreated | ++ | 30 | 223 | 253 | 11.86 | P<0.001 |
| E12 x 143F10 | β-globin x 130 mb chr.7 | α-amanitin | ++ | 38 | 212 | 250 | 15.20 | P<0.001 |
| E12 x 332F3 | β-globin x 73.9 mb chr.7 | untreated | ++ | 20 | 103 | 123 | 16.26 | P<0.001 |
| E12 x 332F3 | β-globin x 73.9 mb chr.7 | α-amanitin | +- | 42 | 208 | 250 | 16.80 | P<0.001 |
| E12 x 263G24 | β-globin x 119 mb chr.7 | untreated | ++ | 27 | 227 | 254 | 10.63 | P<0.001 |
| E12 x 263G24 | β-globin x 119 mb chr.7 | α-amanitin | +- | 36 | 217 | 253 | 14.23 | P<0.001 |
| E12 x 142M15 | β-globin x 117 mb chr.7 | untreated | ++ | 28 | 223 | 251 | 16.60 | P<0.001 |
| E12 x 142M15 | β-globin x 117 mb chr.7 | α-amanitin | +- | 13 | 125 | 138 | 9.40 | P<0.01 |
| E12 x 455N12 | β-globin x 121.45 mb chr.7 | untreated | ++ | 24 | 227 | 251 | 9.56 | P<0.001 |
| E12 x 455N12 | β-globin x 121.45 mb chr.7 | α-amanitin | -- | 25 | 225 | 250 | 10.00 | P<0.001 |
