# Supplementary material for: Maintenance of Long-Range DNA Interactions after Inhibition of Ongoing RNA Polymerase II Transcription
Source: PLoS One. 2008 Feb 20;3(2):e1661. doi: 10.1371/journal.pone.0001661 (PMC2243019; doi:10.1371/journal.pone.0001661)
Supplement: Table S3 — Overview of cryo-FISH results in trans for Rad23a (0.03 MB DOC) [file pone.0001661.s003.doc]

# Table S3. Overview of cryo-FISH results *in trans* for *Rad23a*

***Trans*** interactions

| BACs | Loci | **Treatment** | **4C** | **Overlapping** | **Apart** | **N** | **% overlap** | G-test |
| --- | --- | --- | --- | --- | --- | --- | --- | --- |
| A15 x 32C19 | Rad23a x 118,2 mb chr. 7 | untreated | -- | 3 | 247 | 250 | 1.20 |  |
| A15 x 32C19 | Rad23a x 118,2 mb chr. 7 | α-amanitin | -- | 0 | 257 | 257 | 0.00 |  |
| A15 x 311P1 | Rad23a x 102.2 mb chr 11 | untreated | ++ | 13 | 239 | 252 | 5.16 | P<0.001 |
| A15 x 311P1 | Rad23a x 102.2 mb chr 11 | α-amanitin | ++ | 18 | 235 | 253 | 7.11 | P<0.001 |
| A15 x 334N22 | Rad23a x 84 mb chr. 7 | untreated | ++ | 7 | 252 | 259 | 2.70 | P<0.05 |
| A15 x 334N22 | Rad23a x 84 mb chr. 7 | α-amanitin | +- | 8 | 244 | 252 | 3.17 | P<0.01 |
| A15 x 199K7 | Rad23a x 119,8 mb chr. 11 | untreated | ++ | 21 | 231 | 252 | 8.33 | P<0.001 |
| A15 x 199K7 | Rad23a x 119,8 mb chr. 11 | α-amanitin | +- | 16 | 237 | 253 | 6.32 | P<0.001 |
| A15 x 534F13 | Rad23a x 76,6 mb chr. 15 | untreated | ++ | 12 | 243 | 255 | 4.71 | P<0.001 |
| A15 x 534F13 | Rad23a x 76,6 mb chr. 15 | α-amanitin | -- | 5 | 250 | 255 | 1.96 |  |
