# Supplementary material for: Maintenance of Long-Range DNA Interactions after Inhibition of Ongoing RNA Polymerase II Transcription
Source: PLoS One. 2008 Feb 20;3(2):e1661. doi: 10.1371/journal.pone.0001661 (PMC2243019; doi:10.1371/journal.pone.0001661)
Supplement: Figure S1 — Foci of the actively elongating form of RNAPII (RNAPII transcription factories) are absent after α-amanitin treatment of fetal liver cells (0.29 MB DOC) [file pone.0001661.s004.doc]

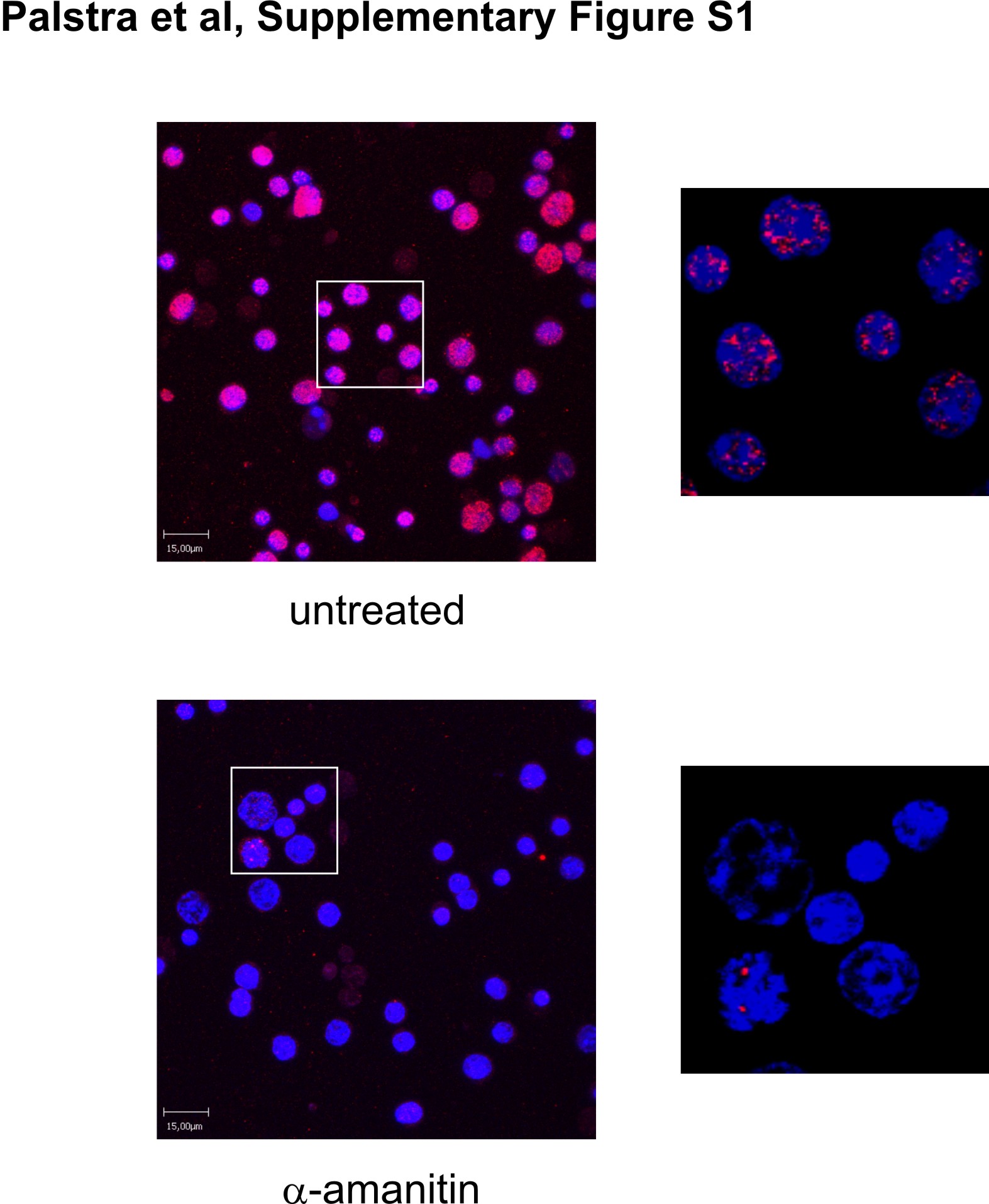


**Figure S1** Foci of the actively elongating form of RNAPII (RNAPII transcription factories) are absent after α-amanitin treatment of fetal liver cells as detected in immune fluorescence microscopy using an antibody against the Ser2 phosphorylated CTD of RNAPII. Top untreated cells, Bottom α-amanitin treated cells. The left panel depicts a compiled stack of confocal images of a whole field of view using a 63x magnification and demonstrates the completeness of α-amanitin treatment. The right panel shows a zoom in of a single confocal stack corresponding to the position of the white box in the left panel and demonstrates the presence of distinct foci of actively elongating RNAPII (RNAPII transcription factories) in untreated cells which are absent in α-amanitin treated cells.
