# Supplementary material for: Maintenance of Long-Range DNA Interactions after Inhibition of Ongoing RNA Polymerase II Transcription
Source: PLoS One. 2008 Feb 20;3(2):e1661. doi: 10.1371/journal.pone.0001661 (PMC2243019; doi:10.1371/journal.pone.0001661)
Supplement: Figure S2 — Clusters of cis-interactions for the β-globin locus (0.76 MB DOC) [file pone.0001661.s005.doc]

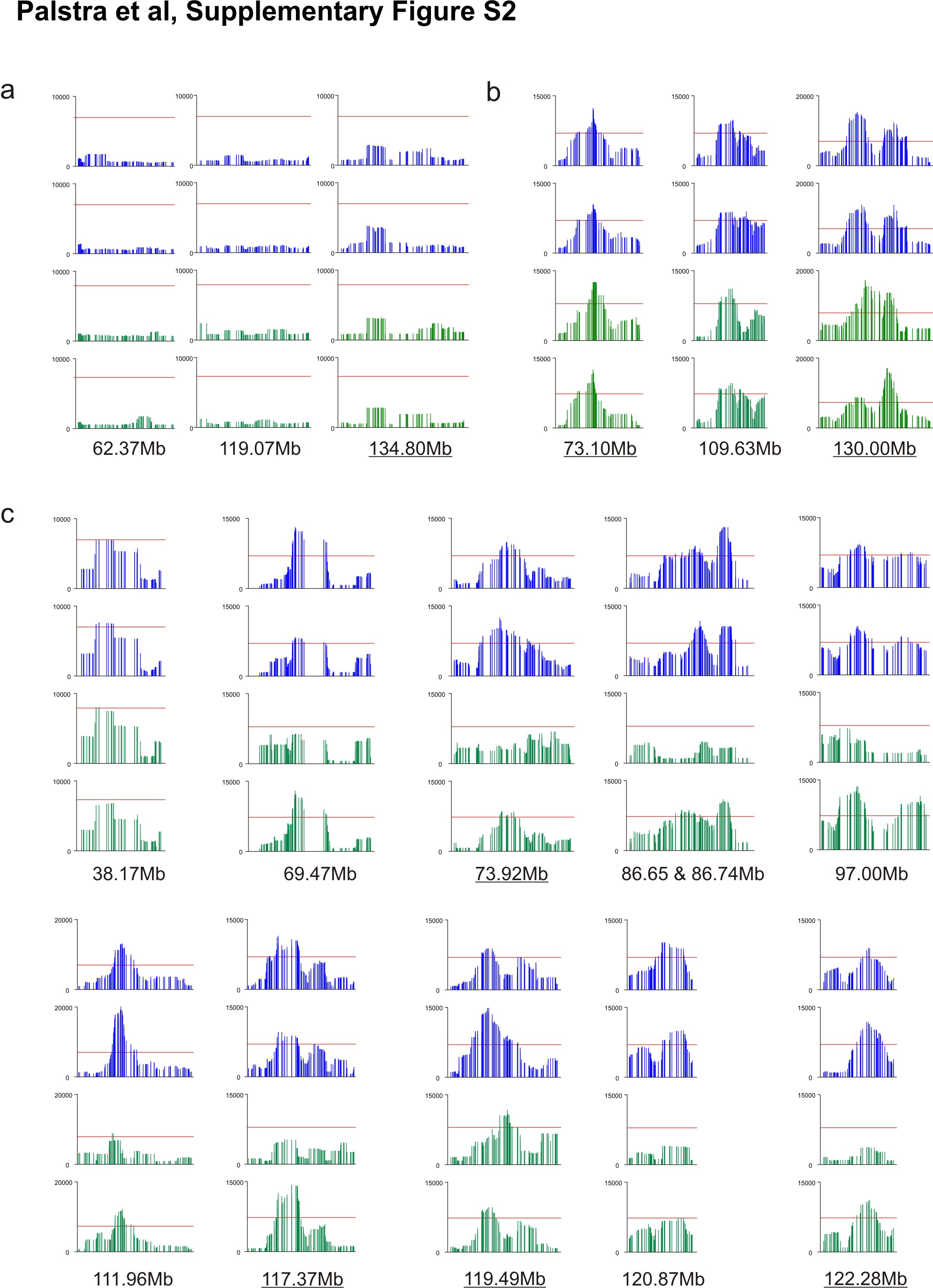


**Figure S2** Clusters of *cis*-interactions for the β-globin locus that are observed in the untreated fetal liver samples (blue) and the α-amanitin treated fetal liver samples (green). (**a**) Running mean data plotted for a selection of three regions that are negative in all samples. (**b**) Running mean data plotted for a selection of three interacting regions in all samples. (**c**) Running mean data plotted for all 11 regions that scored positive in both the untreated samples but only score positive in one of the α-amanitin treated samples. The location of the regions on chromosome 7 is depicted at the bottom of the panels. Underlined regions have been tested in cryo-FISH (see Supplementary information, Table2 online). False discovery rate was set at 5% (red line). Chromosomal positions were based on National Center for Biotechnology (NCBI) build m34.
