# Supplementary material for: Maintenance of Long-Range DNA Interactions after Inhibition of Ongoing RNA Polymerase II Transcription
Source: PLoS One. 2008 Feb 20;3(2):e1661. doi: 10.1371/journal.pone.0001661 (PMC2243019; doi:10.1371/journal.pone.0001661)
Supplement: Figure S3 — Active regulatory sites within the Rad23a locus remain in an active chromatin state after transcription inhibition while inactive regions remain inactive. (0.26 MB DOC) [file pone.0001661.s006.doc]

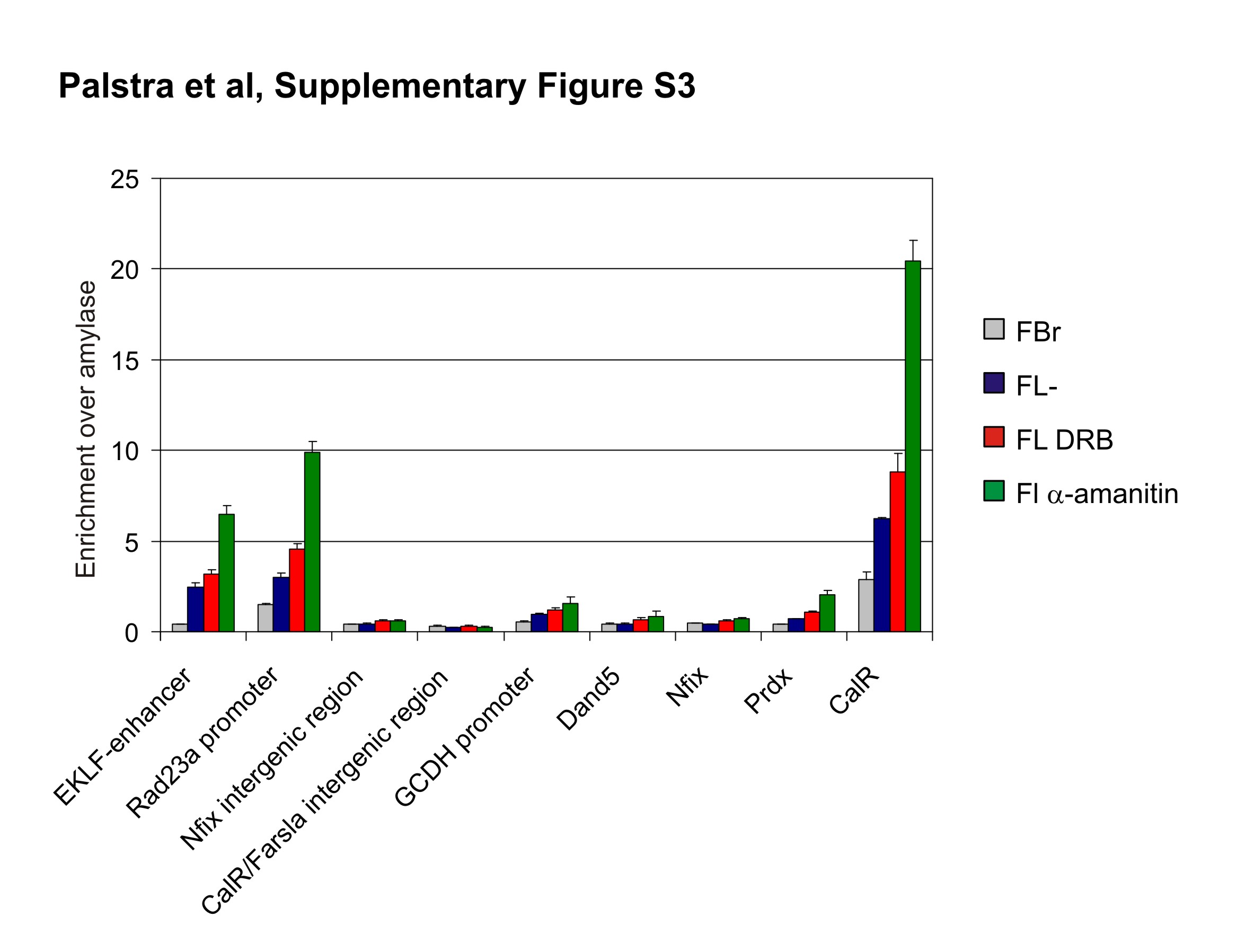


**Figure S3** Active regulatory sites within the Rad23a locus remain in an active chromatin state after transcription inhibition while inactive regions remain inactive. Histone depleted chromatin is detected at regulatory elements of the Rad23a locus using FAIRE. Enrichment is relative to amylase. Grey bars depict fetal brain samples, blue bars depict untreated fetal liver samples, red bars DRB treated fetal liver samples and green bars α-amanitin treated fetal liver samples. Error bars indicate standard error of mean.
