# Supplementary material for: Maintenance of Long-Range DNA Interactions after Inhibition of Ongoing RNA Polymerase II Transcription
Source: PLoS One. 2008 Feb 20;3(2):e1661. doi: 10.1371/journal.pone.0001661 (PMC2243019; doi:10.1371/journal.pone.0001661)
Supplement: Figure S4 — Nucleosome occupancy does not change upon transcription inhibition. (0.21 MB DOC) [file pone.0001661.s007.doc]

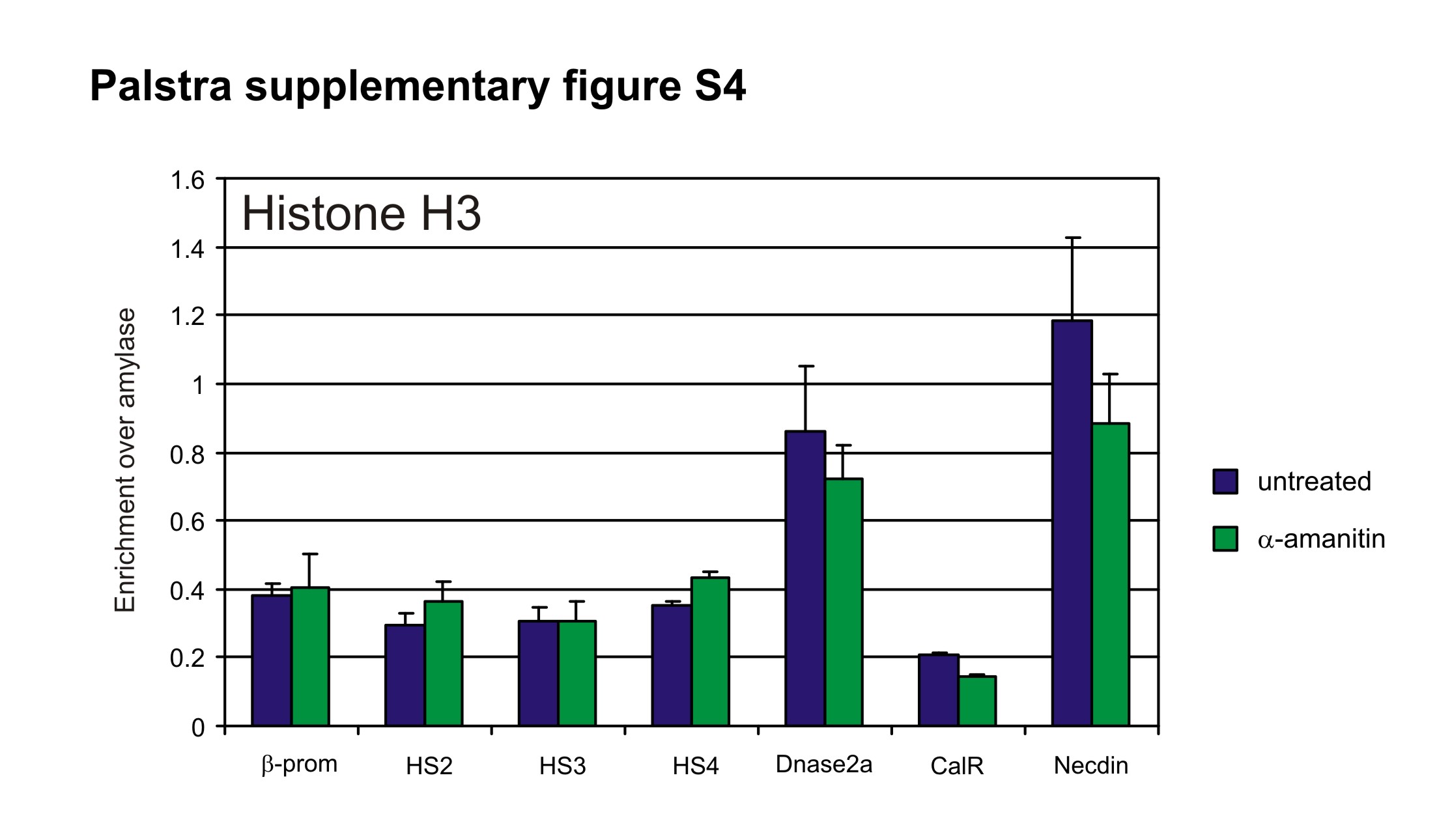


**Figure S4** Nucleosome occupancy does not change upon transcription inhibition. Binding of histone H3 at regulatory sites of the β-globin and Rad23a locus. Enrichment is relative to amylase. Blue bars depict untreated samples, and green bars α-amanitin treated samples. Error bars indicate standard error of mean.
